# The relationship between posttherapeutic Cognitive Behavior Therapy skills usage and follow‐up outcomes of internet‐delivered Cognitive Behavior Therapy

**DOI:** 10.1002/jclp.23403

**Published:** 2022-06-21

**Authors:** Nora Eilert, Rebecca Wogan, Adedeji Adegoke, Caroline Earley, Daniel Duffy, Angel Enrique, Jorge Palacios, Ladislav Timulak, Derek Richards

**Affiliations:** ^1^ E‐mental Health Research Group, School of Psychology Trinity College Dublin Dublin Ireland; ^2^ Clinical Research & Innovation, SilverCloud Health Dublin Ireland

**Keywords:** anxiety, cognitive behavior therapy skills, cross‐lagged panel model, depression, internet‐delivered cognitive behavior therapy

## Abstract

**Background:**

Clients independently applying Cognitive Behavior Therapy (CBT) skills is an important outcome of CBT‐based treatments. The relationship between posttherapeutic CBT skills usage and clinical outcomes remains under‐researched—especially after internet‐delivered CBT (iCBT).

**Objective:**

Explore contemporaneous and lagged effects of posttherapeutic CBT skills usage frequency on iCBT follow‐up outcomes.

**Method:**

Nested within a randomized controlled trial, 241 participants received 8‐week supported iCBT for anxiety and/or depression, completing measures of anxiety, depression, functional impairment, and CBT skills usage frequency at 3‐, 6‐, 9‐, and 12‐month follow‐up. Cross‐lagged panel models evaluated primary aims.

**Results:**

While analyses support a contemporaneous relationship between anxiety, depression, functional impairment, and CBT skills usage frequency, no consistent lagged effects were observed.

**Conclusion:**

Findings align with qualitative research but the role of CBT skills usage in the maintenance of iCBT effects remains unclear. Innovative research modeling temporal and possibly circular relationships between CBT skill usage and clinical outcomes is needed to inform iCBT optimization.

## Key Practitioner Message

- The independent application of Cognitive Behavior Therapy (CBT) skills by clients is an important outcome of all CBT‐based interventions and may contribute to how clinical effects are maintained into follow‐up.
- In this study, participants who reported more frequent use of CBT skills also showed lower symptoms of depression and anxiety and less functional impairment after internet‐delivered CBT had ended.
- However, questions over causal links between posttherapeutic CBT skills usage and clinical outcomes prevail and more robust research is needed to draw firm conclusions.

## 1 INTRODUCTION

Cognitive Behavior Therapy (CBT) encompasses a range of empirically supported psychological interventions addressing cognitive and behavioral processes to bring about behavioral change. CBT interventions vary in length and complexity, demonstrating considerable success in treating a range of psychological issues and symptomology (Butler et al., 2006). An increasingly used format to deliver this therapeutic content is internet‐delivered CBT (iCBT) supported by a clinician, which has been found to achieve treatment effects that are comparable to those in face‐to‐face CBT (Andersson, 2018).

Core outcomes of all CBT‐based interventions are behavioral adaptation and cognitive reappraisal, achieved through teaching clients specific evidence‐based techniques and strategies to self‐manage their difficulties, often referred to as CBT skills (Camacho et al., 2020). These can include behavioral techniques (e.g., activity scheduling, exposure); cognitive techniques (e.g., cognitive restructuring, distancing); acceptance‐focused techniques (e.g., relaxation, mindfulness); and practical coping skills (e.g., problem‐solving, distress tolerance) (Hayes & Hofman, 2018; O'Donohue & Fisher, 2009). To what extent these skills are addressed in CBT‐based interventions depends on the focus, mode of delivery, and length of treatment, with the individual application and implementation of skills likely changing dynamically across different stages of treatment (Camacho et al., 2020).

Despite these differences, the acquisition, use, and practice of these skills by clients is an important outcome of all CBT‐based treatments (Hayes & Hofman, 2018; Hundt et al., 2013). The use of CBT skills has been theorized to be a mechanisms of change in CBT (the compensatory skills theory; Barber & DeRubeis, 1989) implicated in symptom improvements in depression and anxiety during individual, group‐based, and iCBT (Hawley et al., 2017; Terides et al., 2018; Webb et al., 2019). Moreover, skills usage has been proposed to mediate initial change during treatment as well as contribute to the enduring effects of treatment after it has ended (Hollon et al., 2006; Strunk et al., 2013). In particular, greater CBT skills practice postintervention has previously been linked to lower relapse rates and better depression and anxiety outcomes at follow‐up (Michalak et al., 2008; Morgan et al., 2014; Powers et al., 2008). Qualitative studies provide evidence for idiosyncratic benefits of CBT skills usage at follow‐up (French et al., 2017; Glasman et al., 2004).

However, most research on CBT skills has been conducted in the context of face‐to‐face CBT, while only a handful of studies have explored skills usage in iCBT to date. There is now some research to suggest that increases in CBT skills usage early in treatment lead to reductions in symptoms during later stages of iCBT (Forand et al., 2018) and that iCBT clients do in fact acquire and use CBT skills in productive ways posttreatment (Berg et al., 2020; Eilert et al., 2021; Halmetoja et al., 2014). Beyond the aforementioned qualitative studies, longitudinal quantitative explorations of posttherapeutic CBT skills usage after iCBT are scarce and the specific relationship between posttherapeutic CBT skills usage and follow‐up clinical outcomes, therefore, remains unclear.

One important issue in establishing a relationship between a potential mediator of effects, like posttherapeutic CBT skills usage, and clinical outcomes is temporality—or the ability to reliably show that a potential mediator in fact changes before the outcome (Kazdin, 2007). Here, longitudinal designs are needed. These allow for the repeated measurement of potential mediators and outcomes and thereby the estimation of contemporaneous as well as lagged relationships, that is, relationships between clinical outcomes and CBT skills usage at the same time‐point as well as time‐dependent relationships, in which clinical outcomes at one time‐point are predicted by the clients' CBT skills usage at an earlier time‐point (Orth et al., 2020). These lagged effects in turn provide valuable information regarding the direction of the relationship between CBT skills usage and clinical outcomes at follow‐up and represent an important first step in establishing CBT skills usage as a mediator of effect maintenance after iCBT.

Accordingly, the overall aim of the present study was to explore the relationship between posttherapeutic CBT skills usage and clinical outcomes (i.e., anxiety, depressive symptoms, and functional impairment) after the completion of iCBT treatment and across four follow‐up time‐points (3‐, 6‐, 9‐, and 12‐month follow‐up). Given that previous research has proposed CBT skills usage to contribute to the maintenance of effects in CBT‐based interventions (Hollon et al., 2006; Powers et al., 2008) and previously reported qualitative data suggest that clients perceived helpful impacts of continued CBT skills usage posttreatment (including reduced symptoms; Eilert et al., 2021), we hypothesized that:

Hypothesis 1 Higher frequency of CBT skills usage is associated with lower level of anxiety, depressive symptoms and functional impairment after completed iCBT treatment.

Hypothesis 2 Higher frequency of CBT skills usage at one follow‐up time‐point has a lagged effect on symptom levels at the subsequent follow‐up time‐point, resulting in lower levels of anxiety, depressive symptoms and functional impairment then.

## 2 METHOD

### 2.1 Design

This study was nested within the follow‐up period of a randomized controlled trial assessing the effectiveness and cost‐effectiveness of supported, routinely delivered iCBT for anxiety and depression (Richards et al., 2020). Primary trial analyses confirmed the effectiveness of iCBT, finding large between‐group effect sizes favoring iCBT at posttreatment (*d* = 0.55–0.63) and significant further reductions in symptoms of anxiety and depression from 8‐week to 12‐month follow‐up in the iCBT group. The between‐group effect size for functional impairment was moderate (*d* = 0.35), with maintained effects at 12‐month follow‐up (for details see Richards et al., 2020).

Employing a longitudinal design, the current study utilized clinical outcome data (anxiety, depressive symptoms, and functional impairment) and self‐report data on the frequency of CBT skills usage in cross‐lagged panel models to test possible contemporaneous and lagged effects of between‐person differences in CBT skills usage on clinical outcomes (Orth et al., 2020).

### 2.2 Study setting

This study was conducted within step 2 of the Improving Access to Psychological Therapies program (IAPT) in England. IAPT, a stepped‐care treatment model to increase access to evidence‐based interventions for anxiety or depression, offers low‐intensity treatments at step 2, including guided self‐help, iCBT, or group psychoeducation for mild‐to‐moderate presentations of depression and anxiety. These are supported by specially trained psychological wellbeing practitioners, paraprofessionals with a psychology background, supporting and monitoring clients throughout treatment. Clients are referred through GPs, allied services or self‐referred.

### 2.3 Participants and procedures

All users of step 2 services aged over 18 years were eligible to participate. Eligibility criteria were a score ≥9 on the Patient Health Questionnaire 9‐item scale (PHQ‐9) and/or ≥8 on the Generalized Anxiety Disorder 7‐item scale (GAD‐7) and being deemed suitable for iCBT by a service clinician during screening, defined as willingness to engage with internet‐delivered treatment and internet access. Exclusion criteria were suicidal intent/ideation marked by a score >2 on question 9 of the PHQ‐9, currently undergoing psychological treatment for depression or anxiety, diagnosis of a psychotic illness, and substance abuse. Participants provided their informed consent to participate by electronic signature and are unidentifiable from the data. Participants were free to withdraw consent at any stage, in which case they were omitted from the trial and referred to the IAPT treatment as usual service.

Each participant was assigned a supporter before beginning treatment. Based on their primary diagnosis, each of the 361 participants were allocated to either a depression or anxiety arm, then randomized into iCBT (*n* = 241) or wait‐list control group (WLC; *n* = 120) in a 2:1 ratio. At 8 weeks, both groups completed the outcome measurements online and the WLC began treatment. Thus, only iCBT group participants were included in the current follow‐up study. Participants could access the iCBT platform beyond the 8‐week time‐point, allowing them to use the platform after their supported intervention ended. Routinely collected service data suggested that some participants received support beyond the 8‐week time‐point, which primarily appeared to be due to delayed treatment starts. Among the 241 participants randomized to iCBT treatment, the number of participants who may have been still receiving support at 8 weeks was 127, at 3‐month was 54, and 6‐month was 2. At 3‐, 6‐, 9‐, and 12‐month follow‐ups participants again completed measures of clinical outcomes and CBT skills usage the via the online platform. Participants received financial incentives upon completion of the research measures.

### 2.4 Measures

Primary outcome measures were administered at baseline and 8 weeks, with further intervention‐arm follow‐up at 3, 6, 9, and 12 months. Demographic details (see Richards et al., 2020; for a list) were collected at baseline.

#### 2.4.1 Primary outcome measures

The *PHQ‐9* is a self‐report questionnaire measuring depressive symptoms experienced over the previous 2 weeks and is regularly used in research and as a screening tool in healthcare settings (Kroenke et al., 2001). Participants rate each of the nine items on a 4‐point scale from 0 = “Not at all” to 3 = “Nearly every day.” It discriminates well between depressed and nondepressed individuals using a cut‐off score ≥10, with good reliability (Kroenke et al., 2001). Reliable change in depressive symptoms is defined as a reduction of ≥6 in score (National Collaborating Centre for Mental Health, 2021).

The *GAD‐7* features seven questions evaluating the severity of anxiety experienced over the past 2 weeks (Spitzer et al., 2006). It shows good internal consistency (Cronbach's *α* = 83; Rodebaugh et al., 2008), and good test–retest reliability (Newman et al., 2002). Implementing a cut‐off score ≥8 for clinical anxiety, it is widely used in large‐scale studies and healthcare settings (Clark, 2011). A reliable chang e in symptoms is defined as a reduction in GAD‐7 score ≥4 (National Collaborating Centre for Mental Health, 2021).

The *Work and Social Adjustment Scale (WSAS)* is a valid measure of impaired functioning with sensitivity to treatment change and good reliability (Mundt et al., 2002; Zahra et al., 2014). Five items evaluate impairment to the client's ability to function day to day across work, social life, home life, private life, and close relationships. Clients rate each item from 0 = “Not at all” to 8 = “Very severely.” Scores >10 are considered significant impairment with scores >20 considered moderate‐to‐severe impairment (Mundt et al., 2002).

The *Frequency of Actions and Thoughts Scale (FATS)* consists of 12 items measuring the frequency of adaptive behaviors and thoughts related to CBT (CBT skills) in the past week and has shown sensitivity to change during iCBT (Terides et al., 2016). Scores are computed across overall frequency of CBT skill use and four subscales for specific skills of cognitive restructuring, activity scheduling, rewarding behaviors, and social interaction. Nontechnical language is used, meaning items can be completed independent of having previous exposure to CBT. For example, one item assessing activity scheduling asks clients how often did you “plan a pleasant activity to make you feel better?” or to assess cognitive restructuring, how often did you “stop yourself from thinking unhelpful or unrealistic thoughts?” Clients rate each item from 0 = “Not at all” to 4 = “Almost every day.” The FATS full‐scale ranges from 0 to 48 and shows good internal consistency (Cronbach's *α* = 0.86), as do the subscales (Cronbach's *α* = 0.83–0.74). Indeed, higher scores on the FATS, that is, increased skills usage, were reported following iCBT treatment compared to a control group and higher scores were associated with better treatment outcomes (Terides et al., 2018).

### 2.5 Interventions

The internet interventions included “*Space from Depression*,” “*Space from Anxiety*,” and “*Space from Depression and Anxiety*” delivered on a Web 2.0 platform using media‐rich, interactive content. These interventions have demonstrated effectiveness in treating depression and anxiety (Richards et al., 2020) and adhere to the National Institute for Health and Care Excellence guidelines (NICE, 2009, 2011). Seven CBT content modules were delivered in the order preferred by the participant and, depending on the needs of the patient, additional modules are unlockable by their supporter. The modules include psychoeducation and aim to develop skills like self‐monitoring and cognitive distancing, cognitive restructuring, problem solving, graded exposure, mindfulness, and behavioral activation. Unlockable modules include identification of core beliefs, anger management, relaxation, or worry management (Eilert et al., 2021; Richards et al., 2018). Platform interactive tools encourage participants to practice these skills through mood monitoring, worksheets, and audio meditation exercises. Supporters monitored participants' progress through the 8‐week intervention, sending online reviews every 7–10 days to provide encouragement and feedback on work completed.

### 2.6 Statistical analyses

For the purpose of this study, responses from participants still receiving support from their supporter at a given follow‐up time‐point were excluded and counted as missing. Prevalence and patterns of missing data and missing data mechanisms were explored using descriptive statistics, *χ* ^2^, and *t*‐tests between missing and nonmissing cases and Little's Missing Completely at Random (MCAR) test (Little, 1988).

To explore trajectories of CBT skills usage across follow‐up, a marginal model was used, implementing multiple imputation via multilevel joint modeling (Grund et al., 2016) and an unstructured covariance structure. Bonferroni‐adjusted consecutive paired comparisons based on estimated marginal means were employed to further assess differences in the frequency of skills usage between follow‐up time‐points, where applicable.

Path‐analysis via structural equation modeling was used to evaluate cross‐lagged panel models of the effect of CBT skills usage as measured by the FATS on clinical outcomes (PHQ‐9, GAD‐7, and WSAS) at individual time‐points and overall. As proposed by Orth et al. (2020) for each clinical outcome, variable models were built sequentially and nested models were compared via the scaled difference *χ* ^2^ test statistic proposed by Satorra and Bentler (2010). In particular, beyond modeling autoregressive relationships between time‐points for each outcome variable and the FATS, lagged and contemporaneous effects of the FATS on clinical outcomes were included in nested models. Directionality of any relationships between the FATS and clinical outcome variables were assessed via alternative models to explore contemporaneous and lagged effects of the clinical outcome variables on the FATS. Missing data in these models was handled via Full Information Maximum. Likelihood procedures and robust standard errors were implemented in final models (Yuan & Bentler, 2000). Model fit was evaluated via the Model *χ* ^2^, the Comparative Fit Index (CFI), the Root Mean Square Error of Approximation (RMSEA), and its *p*‐value. Overall contemporaneous and lagged effects, that is, the product of individual relationships between time‐points, were calculated where appropriate.

To strengthen the credibility of any potential significant findings in the path‐analysis and given the novelty of the FATS as a research measure, confirmatory factor analyses were conducted to ensure that the constructs measured by the FATS and the PHQ‐9, GAD‐7, and WSAS, respectively, were in fact distinct and did not overlap. For this purpose, three two‐factor models, in which standardized FATS and outcome scale items (PHQ‐9, GAD‐7, and WSAS) loaded onto separate factors of CBT skills usage, depression, anxiety, and impaired functioning, respectively, were evaluated in terms of factor loadings, modification indices, and fit indices.

## 3 RESULTS

A total of 206 participants who completed follow‐up measures at least at one follow‐up time‐point and were not receiving support anymore at this time‐point were included in this study. Among these, the average age was 32.76 (SD 12.59), 153 (74%) identified as female, 162 (79%) as White British, and 180 (87%) as heterosexual. For 107 (52%) anxiety was their primary presentation at service intake and for 99 (48%) it was depression. Among 186 participants who completed outcome measures at 3‐month follow‐up, 104 (50%) had achieved reliable change on the PHQ‐9 (a score reduction ≥6) and 117 (57%) had achieved reliable change on the GAD‐7 (a score reduction ≥4) at 3‐month follow‐up.

Data were deemed to be missing at random. Across the entire treatment group (*N* = 241) and all four time‐points, 29.9% of FATS and 25.6% of PHQ‐9, GAD‐7, and WSAS data were recorded as missing. At 3‐month follow up 23.2%, at 6‐month 25.3%, at 9‐month 38.6%, and at 12‐month 32.4% of FATS data were missing. Little's MCAR test confirmed that data were missing completely at random (*χ* ^2^_(10)_ = 16.25, *p* = 0.093). *χ* ^2^ tests and *t*‐tests comparing those with and without missing FATS data across time‐points were not significant for age (*t*_(960)_ = 1.39, *p* = 0.164), ethnicity (*χ* ^2^_(1)_ = 1.25, *p* = 0.264), sexual orientation (*χ* ^2^_(1)_ = 2.53, *p* = 0.111), psychoactive medication use (*χ* ^2^_(1)_ = 2.64, *p* = 0.104), employment status (*χ* ^2^_(1)_ = 0.98, *p* = 0.323), primary diagnosis (anxiety or depression; *χ* ^2^_(1)_ = 0.57, *p* = 0.452), baseline PHQ‐9 (*t*_(714)_ = −0.49, *p* = 0.626), GAD‐7 (*t*_(713)_ = −0.71, *p* = 0.475), or WSAS scores (*t*_(714)_ = 0.01, *p* = 0.995) but did, however, suggest that more males than females were missing data across follow‐up (*χ* ^2^_(1)_ = 18.57, *p* < 0.001).

**Figure 1:**
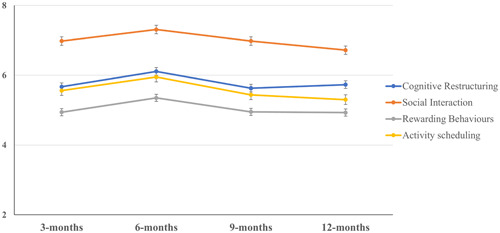
Observed means with standard error bars of FATS subscales across follow‐up time‐points. Three months *n* = 185; 6 months *n* = 180; 9 months *n* = 148; 12 months *n* = 163.

No significant difference in FATS scores between time‐points were found in the marginal model. See Table 1 for model coefficients and total FATS estimated marginal means and Figure 1 for mean FATS subscale scores across time‐points.

**Table 1:** Pooled marginal linear model fixed effect estimates and total FATS estimated means across follow‐up

Effect	*b*	SE *b*	95% CI	Estimated mean (95% CI)
Intercept	23.24	0.67	21.93, 24.56*	23.2 (21.6, 24.9)
6 months	1.30	0.67	−0.02, 2.63	24.6 (22.8, 26.3)
9 months	−0.15	0.71	−1.53, 1.24	23.1 (21.4, 24.8)
12 months	−0.50	0.70	−1.88, 0.88	22.7 (20.9, 24.6)

*Note*: Intercept represents FATS scores at 3‐month follow‐up. Estimates pooled over 100 imputed datasets according to Rubin's rules.

Abbreviation: FATS, Frequency of Actions and Thoughts Scale.

* *p* < 0.0001.

**Figure 2:**
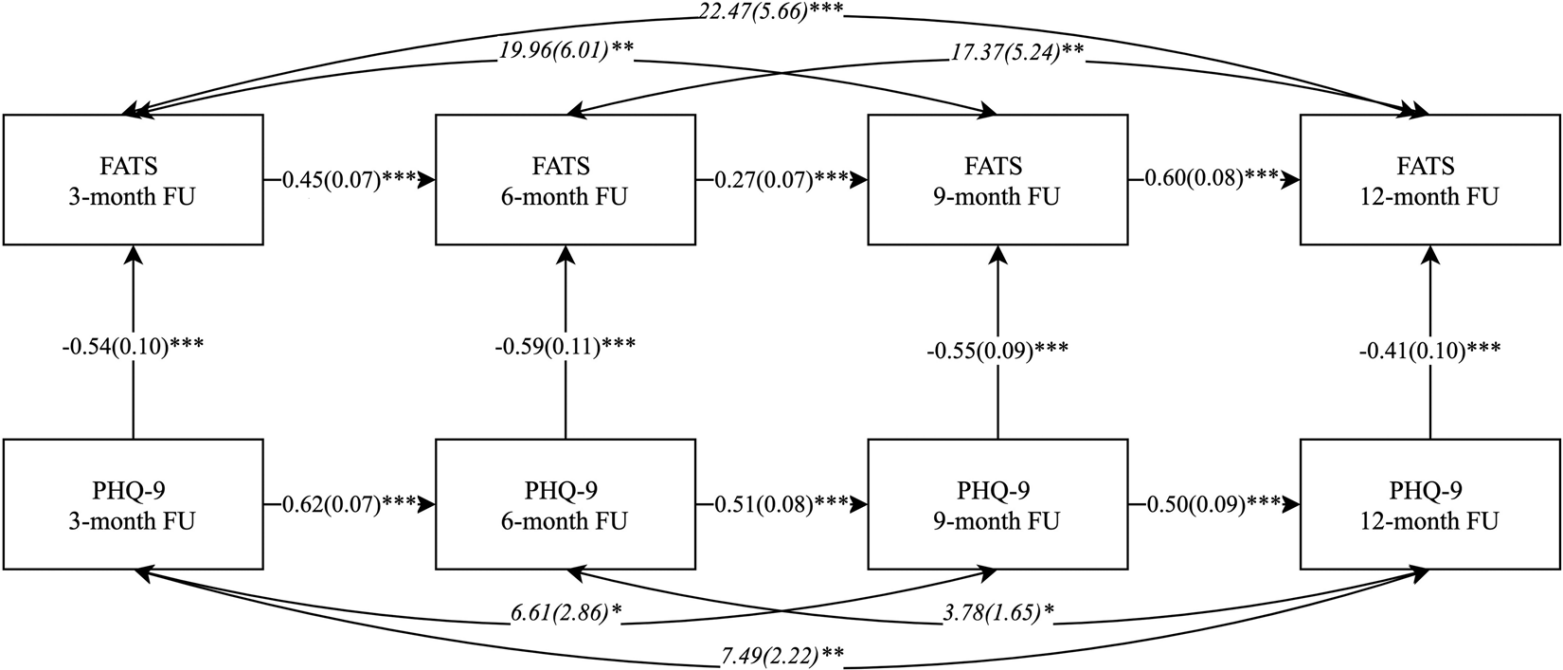
Cross‐lagged panel model of the relationship between FATS and PHQ‐9 across follow‐up. PHQ‐9, Patient Health Questionnaire. FATS, Frequency of Actions and Thoughts Scale. FU, Follow‐up. Single headed straight arrows represent regression coefficients. Double headed curved arrows represent covariances. Standard errors are given in brackets. ***p < 0.001, **p < 0.01, *p < 0.05.

**Figure 3:**
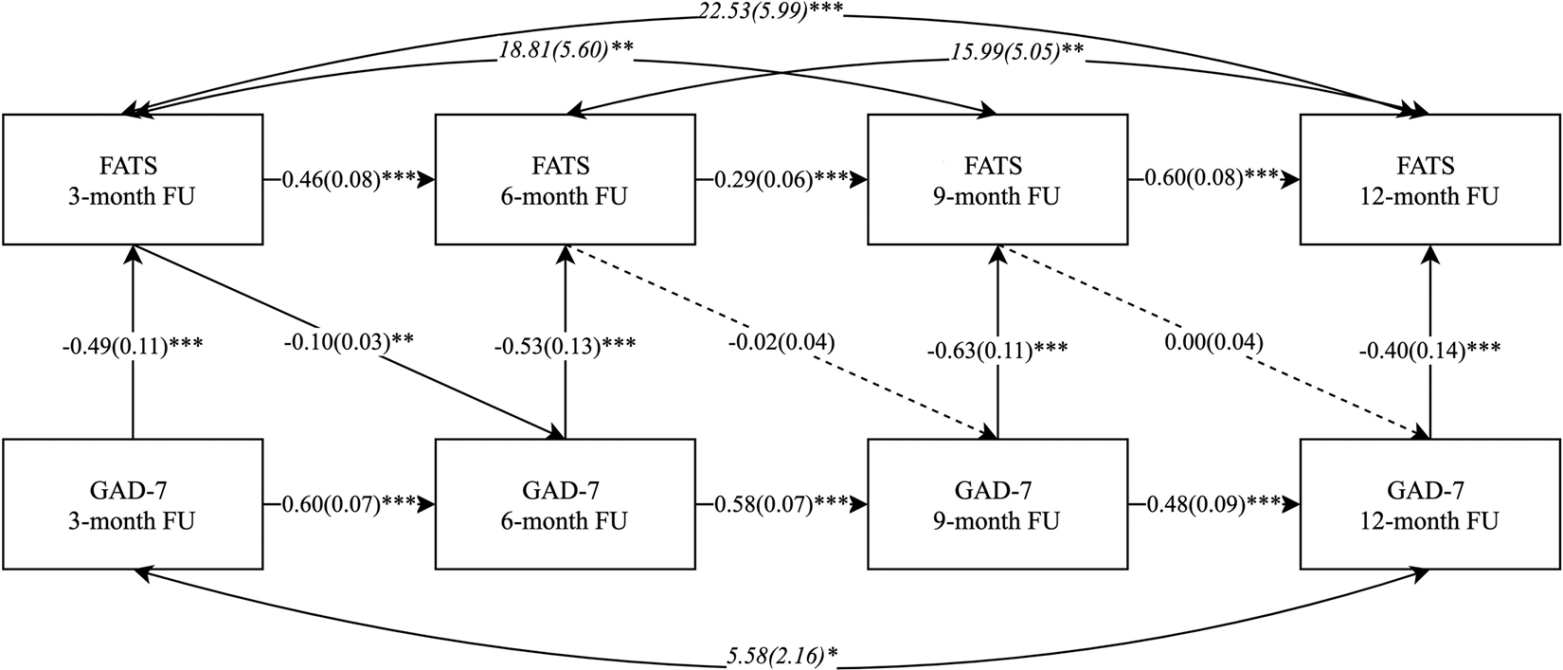
Cross‐lagged panel model of the relationship between FATS and GAD‐7 across follow‐up. GAD‐7, Generalized Anxiety Disorder 7‐item. FATS, Frequency of Actions and Thoughts Scale. FU, Follow‐up. Single headed straight arrows represent regression coefficients. Double headed curved arrows represent covariances. Standard errors are given in brackets. ***p < 0.001, **p < 0.01, *p < 0.05.

**Figure 4:**
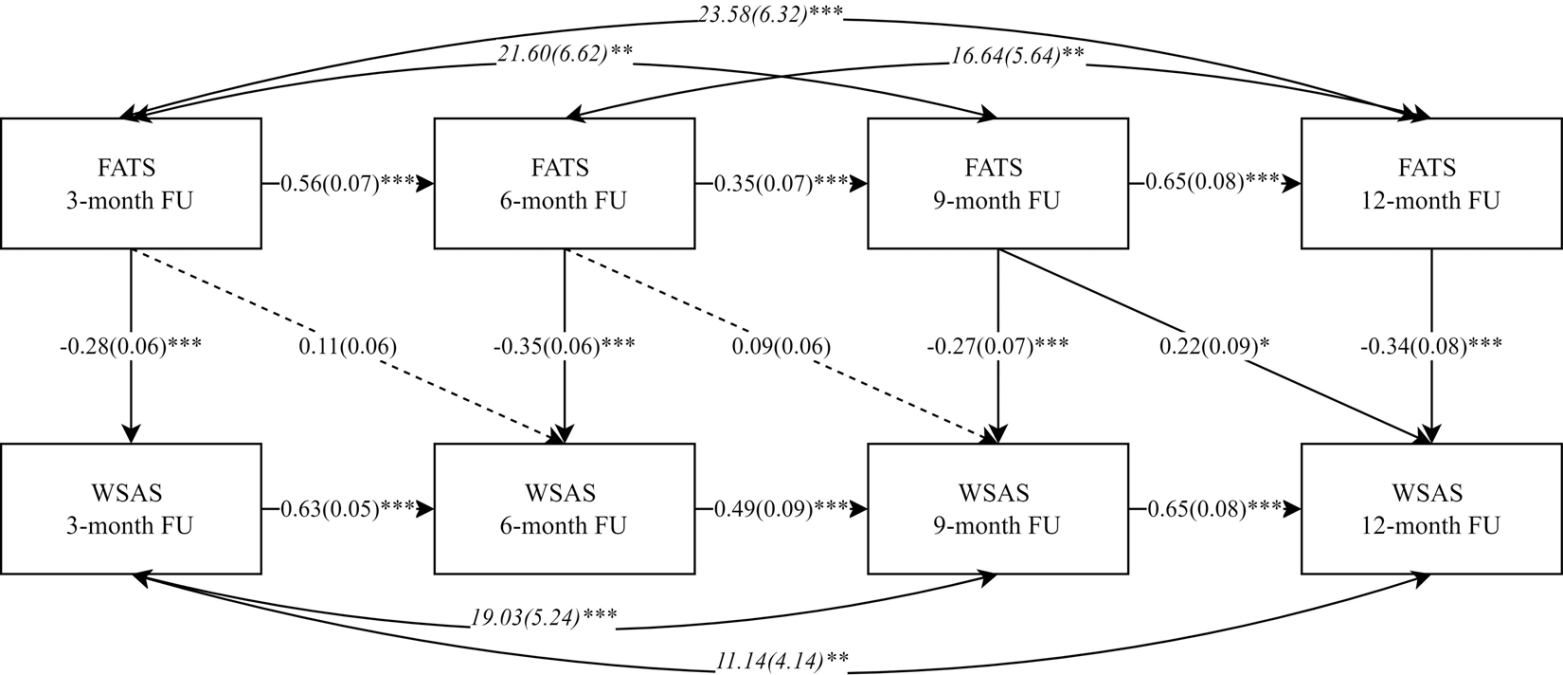
Cross‐lagged panel model of the relationship between FATS and WSAS across follow‐up. WSAS, Work and Social Adjustment Scale. FATS, Frequency of Actions and Thoughts Scale. FU, Follow‐up. Single headed straight arrows represent regression coefficients. Double headed curved arrows represent covariances. Standard errors are given in brackets. ***p < 0.001, **p < 0.01, *p < 0.05.

Three path‐analysis models were built to explore the relationship between the FATS and PHQ‐9, GAD‐7, and WSAS over time (Figures 2, 3, 4). In all three models, the inclusions of paths between the FATS and clinical outcomes beyond the autoregressive paths between time‐points resulted in significantly better fitting models (scaled difference *χ* ^2^ tests *p* < 0.0001); however, the direction and specifics of these paths differed by clinical outcome variable. In relation to depression outcomes, the most parsimonious best fitting models (*χ* ^2^_(12)_ = 16.37, *p* = 0.17; CFI = 0.99; RMSEA = 0.05, *p* = 0.41) included contemporaneous paths from PHQ‐9 to FATS across the four time‐points (*b* = −0.41 to −0.59, SE = 0.09–0.11, *p* < 0.0001; see Figure 2 for details), suggesting that lower depressive symptoms at a given time‐point predicted more frequent use of CBT skills at this time‐point. The overall contemporaneous effect of PHQ‐9 on FATS across time‐points was also significant (*b* = 0.07, SE = 0.03, *p* = 0.018).

In terms of anxiety, a similar picture emerged in relation to the direction of the contemporaneous effect, with lower GAD‐7 scores predicting more frequent use of CBT skills at a given follow‐up time‐point (*b* = −0.40 to −0.63, SE = 0.11–0.14, *p* < 0.0001; see Figure 3 for details). Differently than in the depression model, the best fitting anxiety model (*χ* ^2^_(11)_ = 11.12, *p* = 0.43; CFI = 0.998; RMSEA = 0.02, *p* = 0.75) also included a lagged effect of the FATS on GAD‐7, with higher frequency of CBT skills usage at 3‐month follow‐up predicting lower levels of anxiety symptoms at 6‐month follow‐up (*b* = −0.10, SE = 0.03, *p* = 0.003). This lagged effect did not reach significance at the other time‐points though (see Figure 3 for details). The overall contemporaneous effect of GAD‐7 on FATS across time‐points (*b* = 0.06, SE = 0.03, *p* = 0.063), and the overall lagged effect of FATS on GAD‐7 were insignificant (*b* = 0.00, SE = 0.00, *p* = 0.945).

Path‐analysis of the relationship between impaired functioning and CBT skills usage at follow‐up suggested a contemporaneous effect of FATS on WSAS, with higher frequency of CBT skills usage predicting lower levels of impaired functioning at all follow‐up time‐points (*b* = −0.27 to −0.35, SE = 0.06–0.09, *p* < 0.0001; see Figure 4 for details). In addition, the final model exhibiting best model fit (χ^2^_(11)_ = 7.53, *p* = 0.67; CFI = 1.0; RMSEA = 0.00, *p* = 0.85) also included a lagged effect of FATS on WSAS. While only one of the three possible lagged effects reached statistical significance, 9‐month FATS scores significantly predicted 12‐month WSAS scores (*b* = 0.23, SE = 0.09, *p* = 0.02), all three possible lagged effect were positive in valence—meaning that higher frequencies of CBT skills usage at one time‐point were actually related to higher levels of impaired functioning at the subsequent time‐point. See Figure 4 for details. The overall contemporaneous effect of FATS on WSAS across time‐points was significant (*b* = 0.009, SE = 0.005, *p* = 0.047), whereas the overall lagged effect of FATS on WSAS was insignificant (*b* = 0.002, SE = 0.003, *p* = 0.385).

Finally, confirmatory factor analyses confirmed that the constructs measured by the FATS and the respective outcome variables were in fact distinct. All three two‐factor models exhibited good model fit after items from the same scale were allowed to covary (FATS and PHQ‐9 model: *χ* ^2^_(179)_ = 283.32, *p* < 0.001; CFI = 0.952; RMSEA = 0.057, *p* = 0.175; FATS and GAD‐7 model: *χ* ^2^_(142)_ = 186.42, *p* = 0.007; CFI = 0.980; RMSEA = 0.042, *p* = 0.792; FATS and WSAS model: *χ* ^2^_(107)_ = 143.78, *p* = 0.010; CFI = 0.979; RMSEA = 0.048, *p* = 0.558). All individual item factor loadings were significant (factor loadings FATS: 0.53–0.80; PHQ‐9: 0.48–0.84; GAD‐7: 0.75–0.90, and WSAS: 0.70–0.91; *p* < 0.001). Modification indices did not flag any significant factor loadings or covariances of items across the different scales.

## 4 DISCUSSION

The current study set out to explore the relationship between posttherapeutic CBT skills usage and clinical outcomes in the context of routinely delivered iCBT treatment. Utilizing data from a large cohort and collected longitudinally across four follow‐up time‐points, our findings support an association between CBT skills usage and clinical outcomes, with better anxiety, depression, and functional outcomes potentially being related to more frequent skills usage. In this vein, our first hypothesis appeared partially supported, in that the presence of a relationship between skill usage and clinical outcomes was clearly present but the direction of this relationship seemed much less clear. An important finding regarding the relationship between skills usage and clinical outcomes would have been the presence of time‐lagged effects; however, the current study did not detect any reliable and consistent lagged effects of skills usage on clinical outcomes, therefore failing to meet this second hypothesis.

Extending upon the qualitative findings from the same cohort of participants previously reported on by Eilert et al. (2021) and other qualitative studies on follow‐up outcomes of iCBT (Berg et al., 2020; Halmetoja et al., 2014), our findings align with the idiosyncratic benefits (e.g., reduced symptoms, insight, active engagement in skills usage) participants describe in relation to using CBT skills after completing iCBT in those studies. In relation to face‐to‐face CBT, our findings of positive associations between skills usage and clinical outcomes somewhat resonate with previous research (Michalak et al., 2008; Morgan et al., 2014; Powers et al., 2008; Strunk et al., 2013); nevertheless, questions around the direction of this relationship remain. In particular, while the frequency of CBT skills usage predicted the functional impairment in a contemporaneous fashion as expected, frequency of CBT skills seemed more likely to be contemporaneously predicted by symptoms of anxiety and depression.

Surprising as it is, this finding resonates with qualitative accounts obtained from some of the same participants (reported in Eilert et al., 2021), suggesting that at least some participants purposefully select and combine different CBT skills based on the symptoms they are currently experiencing. Thus, it is possible that the relationship between CBT skills usage and clinical outcomes is a circular one, in which current symptomatology gives rise to skills usage, which in turn then reduces symptoms—something participants reported in the qualitative report also. Here, it would seem plausible that functional impairment, which is by definition secondary to primary symptoms of anxiety and depression, does not give rise to skills usage to the same degree. Nevertheless, these considerations ought to be treated as tentative at best as the absence of any reliable and consistently observed lagged effects in this study—flowing either from CBT skills usage to anxiety, depression and functioning or vice versa—preclude conclusions regarding the temporality of these effects (Kazdin, 2007).

The absence of any consistent lagged effects in the current study may need to be understood in relation to the follow‐up assessment schedule employed, which left 3‐month intervals between the different time‐points when shorter timeframes may be more relevant in this context. Exploring the relationship between CBT skills usage and clinical outcomes during group CBT via daily assessments, Camacho et al. (2020) found lagged effects of CBT skills usage on well‐being and symptom outcomes, highlighting how such designs may be better equipped to model the likely more instantaneous relationship between skills usage and clinical outcomes. Future research would be well advised to consider diary study or even ecological momentary assessment designs (Shiffman et al., 2008) to capture this relationship better and increase the power needed to appropriately evaluate intricate relationships between skills usage and outcomes.

The study is not without limitations, especially in relation to how CBT skills usage was measured. The FATS does not assess perceived helpfulness of skills which has been shown to mediate the relationship between frequency and treatment outcomes previously (Powers et al., 2008). Additionally, it does not account for the quality of skills usage, so whether skills were acquired appropriately or executed effectively. This would appear important given that participants may adapt skills or use them implicitly (Eilert et al., 2021; French et al., 2017; Morgan et al., 2014) and it remains unclear if how much individuals benefit from skills usage is influenced by whether this skills usage is explicit (i.e., formal) or implicit (i.e., informal; Berg et al., 2020; Hollon et al., 2006). Moreover, the FATS does not include items related to mediation or relaxation skills. These are a common component of many CBTs nowadays, were part of the current iCBT interventions, and participants reported benefits from their usage (Eilert et al., 2021), resulting in a potential underreporting of skills usage via the FATS. We also did not measure the FATS at baseline, meaning we could not control for pre‐interventions skills usage or whether skills usage increased during active treatment. As the FATS uses nontechnical language equipped to assess CBT skills usage independent of previous CBT exposure, it is likely that at least some between‐person variance in pretreatment skills usage existed, which in turn could have influenced how much participants benefitted from the acquisition and continued use of skills posttreatment.

Additionally, the level of missing data in the analyses was rather high, limiting power and therefore conclusions we can draw from the analyses. While we did not include responses from participants while they were still within the supported period of the intervention, the fact that not all participants finished active treatment on time is another limitation of the study. Also, implementing cross‐lagged panel models and path‐analysis over more complex longitudinal modeling techniques, we were unable to explore the relationship between within‐person changes in CBT skills usage and clinical outcomes over time or control for measurement error and random variance components (Orth et al., 2020). Finally, as we did not follow up the control group, we cannot say for certain whether CBT skills usage increased as a function of iCBT treatment (as explained above, the FATS uses nontechnical language and it is possible that participants may have been using coping skills resembling CBT skills independently of iCBT).

In conclusion, while the current study provides some evidence that posttherapeutic CBT skills usage and follow‐up clinical outcomes are related, it strongly highlights the need for future research implementing more comprehensive measures of CBT skills usage and more rigorous designs and techniques for modeling lagged and possibly circular effects between skills usage and anxiety and depression outcomes. A better understanding of this relationship will not only have scientific relevance in informing mechanisms of effect maintenance in iCBT but also be valuable in improving upon current iCBT interventions and routine care outcomes by, for example, including booster sessions for those with lapsed skills usage posttreatment or adjunctive face‐to‐face sessions for those who are not acquiring skills appropriately during iCBT.

### ETHICS STATEMENT

The primary trial, including this study, was approved by the NHS England Research Ethics Committee [REC Reference: 17/NW/0311].

### PEER REVIEW

The peer review history for this article is available at https://publons.com/publon/10.1002/jclp.23403.

## Data Availability

Data analyzed herein are available upon request from the corresponding author.
